# A paedophile scan to prevent child sexual abuse in child care? A thought experiment to problematize the notion of alignment in Responsible Research and Innovation

**DOI:** 10.1186/s40504-017-0049-7

**Published:** 2017-02-28

**Authors:** Irja Marije de Jong, Frank Kupper, Corine de Ruiter, Jacqueline Broerse

**Affiliations:** 1 Athena Institute, Faculty of Earth and Life Sciences, VU University Amsterdam, De Boelelaan 1085, 1081 HV Amsterdam, The Netherlands; 2 Forensic Psychology section, Faculty of Psychology and Neuroscience, Maastricht University, Postbus 616, 6200 MD Maastricht, The Netherlands

**Keywords:** Responsible research and innovation, Neuroimaging, Inclusive deliberation, Early closure, Fact/value diversity

## Abstract

Responsible Research and Innovation (RRI) is a science policy concept that gained traction from 2000 onwards in the EU and US, in which alignment on purposes and values between different stakeholders is a key aspect. This thought experiment problematizes this particular notion: ethically acceptable and societally desirable outcomes are not necessarily achieved when alignment is a consequence of early closure. To argue this point, we took the example of the potential development of scanning technology for the detection of paedophilia among job applicants, for which indicators of broad societal support were found in an RRI project on neuroimaging. We analysed this case by looking through several lenses, obtained by structured and non-structured literature searches. We explored how facts and values are masked when a taboo topic is considered. This results in the black boxing of the problem definition, potential solutions and development trajectories. Complex unstructured problems can thus be perceived as manageable structured problems, which can in turn lead to irresponsible policies surrounding technology development. Responsible processes of research and technology development thus require the involvement of a critical reflector who is alert to signs of early closure and who prevents foreclosure of ongoing reflexive deliberation. There is an important role for ethical, legal and societal aspect studies within the framework of RRI. This paper shows that the concepts of “value/fact diversity masking” and “early discursive closure” are new avenues for RRI research.

## Introduction

Responsible Research and Innovation (RRI) is a science policy concept that gained traction from 2000 onwards in the EU and US. It presumes that alignment on purposes and values between the different stakeholders, such as scientists and the public, is a prerequisite for the responsible development and embedding of emerging technologies. Inclusive deliberation, an exchange of ideas among a wider set of stakeholders representing a diversity of perspectives, in early phases of technology development is seen as essential to such an effort (Owen et al. 2012). However, as the concept of RRI is still emerging, it remains largely unclear how proper alignment can be realized in practice. We surmise that in the emerging RRI literature, the concept of alignment is not examined critically to a sufficient extent.

One of the added values of the concept of RRI is that it recognizes the importance of coupling inclusive deliberation with policy and decision-making processes (Owen et al. 2012). The underlying thought is that inclusive deliberation can uncover the range of relevant values to be taken into account in the technology development and subsequent implementation phases. Moreover, inclusive deliberation activities are thought to aid in attaining alignment between the diversity of values deemed important by the different stakeholder groups, thereby providing important steps towards legitimate policy choices. However, alignment can also be a symptom of what we call ‘early closure’, which is problematic as it forecloses continued reflexive deliberation. Before we delve deeper into the concept of early closure, we first have to take a closer look at what constitutes alignment.

**Fig. 1:**
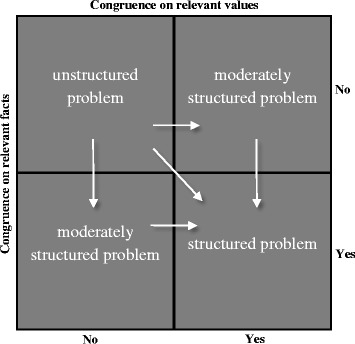
Four types of policy problems and the effect of biases. Adapted from Hisschemöller and Hoppe (1995)

One early manifestation of alignment is the existence of a shared vision of the problem at hand. In the work of Hisschemöller and Hoppe (1995) four types of policy problems are discerned via the juxtaposition of two separate dimensions: congruence and incongruence on the relevant ‘facts’ of the problematic situation versus congruence and incongruence on the relevant ‘values’ (see Fig. 1). The resulting policy problems are structured problems (congruence on relevant facts and values), moderately structured problems (congruence on either relevant facts or relevant values) and unstructured problems (incongruence on both the relevant facts and values). Structured problems are the easiest to solve as there is consensus on which values should be respected or optimized by the solution, as well as consensus on the what resources are necessary for solving the problem. The process of alignment between different stakeholders can thus yield problems that are ‘doable’. Unstructured problems are the hardest problems to solve, because of their high uncertainty and highly political character. In fact, this type of problem requires problem structuring through deliberation efforts, rather than straightforward problem solving strategies. Hisschemöller and Hoppe (1995) also convey that value diversity can be hidden from view when certain stakeholders representing a different perspective are not seen as ‘legitimate’ participants of the policy making process. Uncertainties on relevant facts can be masked when not all arguments are regarded as pertinent, for example when a policy elite manages to frame a certain problem exclusively within an economic rationale. These biases constitute a masking of value conflicts or uncertainties on relevant facts, which causes an unstructured problem to be perceived as a (moderately) structured one. This has happened in the past for the siting of hazardous waste treatment facilities in the Netherlands and the US. In the Dutch case, the siting of the hazardous waste treatment facility was framed as a structured problem, by excluding citizen participation on grounds of their ‘irrationality’, thereby allowing experts to treat it as a technical problem (Hisschemöller and Midden 1989). In the case of the US, the problem was framed as moderately structured, by framing the concerns of citizens in purely economic terms, and discarding other environmental or health concerns. In other words, efforts can be undertaken to solve alleged (moderately) structured problems via a technocratic strategy foregoing the more fundamental value-laden issues. This strategy is not likely to result in ethically acceptable and societally desirable outcomes. In fact, this is how intractable controversies may arise (Hisschemöller and Hoppe 1995).

Suppression of fact and value diversity results in underdetermination of the societal problem at hand, which is problematic for those concerned with responsible development and embedding of technologies. If a process of inclusive deliberation does not encompass reflexivity with respect to the entire range of associated values in an RRI process, a too simplistic view of the problematic issue is likely to emerge. The model by Hisschemöller and Hoppe (1995) informs us that this will be perpetuated in the policy choices being made, which will decrease the chance of an ethically acceptable outcome.

We discovered that this problem is not just hypothetical but a very real one during our wider research project on the responsible development and societal embedding of emerging neuroimaging technologies in Dutch society.^1^ We became aware of potential masking of fact and value diversity in relation to potential neurotechnology applications for the detection of paedophilic sexual interests. We held six focus group discussions with randomly chosen citizens on ideas, opportunities and concerns regarding neuroimaging applications (Arentshorst 2014). Often citizens raised the topic of paedophilic interest identification as an opportunity for neuroimaging applications: rarely was this technological option deemed problematic. However, in relation to *medical* applications of neuroimaging, participants put forward several problematic aspects, such as reliability and the privacy of their thoughts. It was not only citizens who raised the topic of paedophilia in relation to neuroimaging. One third of interviewed scientists employing neuroimaging technologies for research purposes relevant to justice and security (total *n* = 20) brought up this subject as well, despite non-involvement in research on this topic. Some neuroscientist saw clear opportunities, while others raised the topic because of associated concerns, for example the likelihood that stereotypes about paedophiles are perpetuated in neuroimaging research. One remarkable example in previous work (de Jong et al. 2015) concerned a scientist who employed neuroimaging technologies who seemed to make an exception for the special case of paedophilia when talking about stringent criteria for the reliability and validity of neuroimaging technologies used in criminal justice:

*“I always think it is worse when someone is put behind bars while innocent [because of the technology] than the other way around [a criminal walks free]. However, when dealing with paedophiles, you might have another opinion about this.”*

These examples show that issues of reliability, privacy and civil liberties can be overruled when it concerns the target population of paedophiles, suggesting a potential suppression of value diversity. The risk of perpetuation of paedophile stereotypes in neuroimaging research is also indicative of a lurking low diversity of relevant facts regarding characteristics of paedophiles. This could be due to the fact that paedophilia is a taboo topic. Although very little stigma research has been performed on the subject of paedophilia, preliminary evidence suggests that paedophilia is currently among the most stigmatized human characteristics in the Western world (Goffman 1963, Jahnke and Hoyer 2013). Reactions to paedophiles are particularly negative, and social distance (Link et al. 1999) is high. The history of sexuality research has shown that scientific research activities are linked to societal conceptualisations of sexuality and proper conduct and that scientific findings can be used to back up pre-existing prejudices (Wolpe 2004).

We argue that the future application of neuroimaging technology for the detection of paedophilia could function as an example of how the masking of value and fact diversity can be induced from the earliest stages onwards, thereby yielding early closure. Note that a functional paedoscan as such does not yet exist. However, the earlier findings indicate that paedoscan ‘imaginaries’ (Nordmann 2010) seem to be present, as wishes to be fulfilled or a potential to be realised in the future, but with the wish or potential contained in the now. Although the paedoscan does not exist yet, imaginaries can already shape relationships and can be enacted in everyday practices. Therefore, this thought experiment is relevant in the absence of an applied technology. The problematic situation surrounding the potential paedoscan appears as a (moderately) structured problem, or in other words, as a doable problem with broad support on the way to solve it. This will allow us to explore whether this type of alignment is indeed an obstacle for a genuine and open dialogue on the problematic issues concerned. The aim of this paper is to problematize the notion of alignment and to draw lessons from the phenomenon of early closure for the emerging framework of RRI.

As our wider research project focuses on the Netherlands, we draw from the Dutch context in shaping this case study of the Dutch ‘paedoscan’. However, we will also draw from the larger international literature as technological and scientific developments surrounding paedophilia and child sexual abuse are not confined by borders, and as societal reactions to this controversial issue in the Netherlands appear to be comparable to those observed in other (Western) countries, although comparative research on attitudes and affective reactions towards people with pedophilia remains scarce (Jahnke and Hoyer 2013, Silverman and Wilson 2002 ).

## Case description

In the Netherlands, brain-based detection of paedophilia came into focus in the media in 2011. A Dutch neuroscientist raised the question whether a recent major scandal^2^ in an Amsterdam-based child day care centre in 2010 could have been prevented by the use of ‘paedoscans’ for applicants for work at child day care facilities (Kist 2011, Lamme 2012). Lamme referred to the work of sexologist and neuroscientist Jorge Ponseti, who claimed the ability to accurately assess sexual preference based on a comparison of functional Magnetic Resonance Imaging (fMRI) brain scans of 24 self-reported paedophiles and 32 heterosexuals (Ponseti et al. 2012). The experiment was based on the idea that brain activity patterns of paedophiles would differ from those of heterosexual males, when confronted with pictures of naked children or nude adults. fMRI can visualise the structure, function and metabolism of the intact and living brain, although it does not have direct access to the inside of the skull. The technology makes inferences about brain activity by measuring changes in cerebral blood flow and the resulting ‘pictures’ of the brain are construed through complex statistical analysis of data.

Some scientists objected to this proposal in Dutch newspapers and scientific blogs, raising ethical concerns and arguing that the diagnostic accuracy of fMRI on an individual level is still too low. For example:

*“You can have all kinds of dubious preferences and interests, but that does not mean that you act upon it. This is especially relevant for paedophilia, because research shows that there are people with a sexual preference for children who nevertheless still keep that under control” (ten Broeke* 2011 *).*

The media attention reached an advisory body of the Dutch government, and a meeting was organized to explore, among others, the case of the paedoscan (MoSJ 2012). One of the conclusions of this meeting was that more insight was needed into dealing with potential societal demand for the application of the paedoscan. Neuroscience can aid in yielding increased understanding of sexual behaviour and, potentially, in providing future options to identify and possibly modulate destructive sexual behaviours (e.g. Renaud et al. 2011). However, the potential of neuroimaging technologies also raises ethical discussions about their consequences, intended as well as unintended. Neurobiological screening for paedophilia is extremely sensitive considering the exceptionally stigmatised label of paedophiles (Goffman 1963, Jahnke and Hoyer 2013). Besides the taboo of paedophilia, the paedoscan also touches upon taboos of sex offending and childhood sexuality (Angelides 2004).

For the purpose of this thought experiment, we will start with traces of the prospective paedoscan found in Dutch media. This means that we consider as characteristics the development of neuroimaging technology to prevent child sexual abuse by screening for the source of prospective sexually deviant behaviour among job applicants for positions in child care institutions. Our research question is. We will extrapolate these traces by examining relevant scholarly and scientific literature concerning the technology, society and the envisioned context of application: the child care institutions. Therefore, our central research question is: in what ways may the masking of fact and value diversity interfere with problem structuring in the societal discussion of neurotechnology applications for the detection of paedophilic sexual interests among job applicants?

## Methodology

A thought experiment is a creative process in which the researcher playfully generates potential linkages and relationships (Schram 2006). As we are dealing here with a prefactual thought experiment, extrapolating a potential future from a certain present, our starting point was the identification of established knowledge in literature, from which we developed new significance and questions. To this end, we performed both a structured and an unstructured literature review. We executed a systematic search through the scientific literature to map the relevant developments regarding the technological side of this case study. The systematic search strategy was adopted here as it allowed us to make a structured analysis of the scientific system producing these scientific articles. For the argumentative side of this case study, we performed a non-systematic search through the scholarly literature with an iterative approach, as this enabled us to explore more complex dynamics.

### Systematic literature search and analysis

**Fig. 2:**
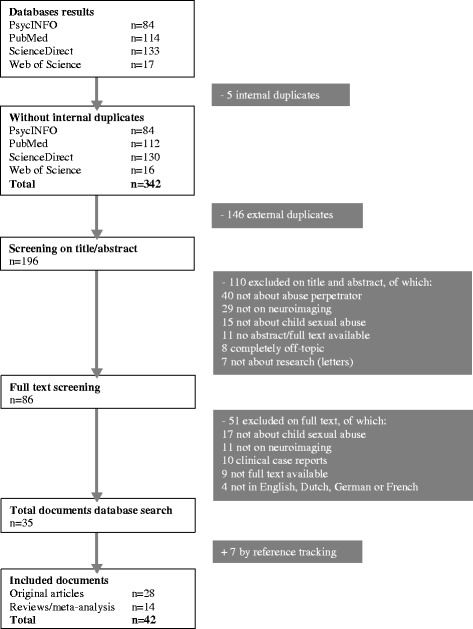
Flow chart of the systematic search of the literature

A systematic literature search strategy was adopted to find scientific literature on neuroimaging research on perpetrators of child sexual abuse. We searched the databases Web of Science, PubMed, PsycINFO and ScienceDirect on 29 or 30 October of 2013. The building blocks of the queries per database are specified in the Appendix. Inclusion and exclusion criteria are displayed in Table 1. The systematic search resulted in 28 original data articles and an additional 14 review/meta-analysis articles. The flow chart with the inclusion and exclusion criteria is displayed in Fig. 2. The sources were coded using qualitative analysis software (MAXQDA). The analytic categories are displayed in Table 2.

**Table 1:** Inclusion and exclusion criteria for the systematic literature search

	Inclusion criteria	Exclusion criteria
*Subject*	paedophile or suspected/convicted/self-proclaimed perpetrator of child sexual offence	victim of child sexual offence, perpetrators of non-sexual crimes, people with other paraphilia
*Method*	adoption of neuroimaging technology to measure the subject	neuroimaging technology is mentioned but not adopted to measure the subject
*Article type*	original research, meta-analysis, review	editorial articles, letter to the editor, clinical case reports
*Availability*	full-text accessible to the authors	full-text not accessible to the authors
*Language*	English, French, Dutch, German	others

**Table 2:** Analysis categories for neuroimaging research on child sexual abuse offenders

Neuroimaging modality (CT/EEG/MRI/fMRI/PET)	
Research interest (sexual deviancy, incest, child sex offending, paedophilia)	
Scan type (e.g. brain mechanism, assessment, general, brain damage, brain differences, diagnosis or identification, neurofeedback)	
Research subjects (e.g. paedophiles (self-declared, phallometrically established, psychiatrically established), (healthy or matched) control group, child sex offenders, nonsexual offenders, nonviolent nonsexual offenders, violent sex offenders, incest offenders, students and academic staff)	
Setting (legal, forensic, clinical forensic, clinical, lab/research)	
Nature of problem statement (e.g. clinical problem, societal problem, legal problem, scientific problem)	
Prospective relevancy (clinical forensic relevancy, scientific relevancy, societal relevancy, clinical relevancy)	

### Non-systematic search of the scholarly literature and analysis

For the non-systematic search through the scholarly literature we used Google Scholar. Search keys related to paedophilia, neuroimaging research, child sex abuse and entrusted care, as well as possible dynamics within certain societal spheres, such as social policy, politics, society and media. The search was not restricted to a certain time period. For recent developments in social policies, we also looked for news articles for which the databases LexisNexis Academic database and Google were used. The identified sources were read and phenomena relevant to the masking of fact or value diversity were noted down in a categorization matrix. Examples of phenomena were tough social policies for sex offenders, taboo, disgust, political capital, lobby, moral panic, workplace screening regulation and mediatisation (the process through which media shapes and frames the disccourse within the political and societal realm, see Lilleker 2006). Based on this matrix, overarching themes were constructed in conversations between the first and second author. Thematic data-analysis (Braun and Clarke 2006) took place according to these five themes (see Table 3) and was performed by hand.

**Table 3:** Thematic analysis categories for the scholarly literature

1. Definitions of child sex abuse and paedophilia	
2. Views on neuroimaging research	
3. Discursive space in the media and the political arena	
4. Social policy	
5. Institutional context of child care	

## Results

We found that the development of technologies to prevent child sex abuse between those working in formal care settings and the children placed in their care, is susceptible to the masking of fact and value diversity, resulting in a lack of critical reflection on – or the black boxing^3^ of – the problematic situation. In this section, we will show that: 1) black boxing can influence societal problem definitions of the relation between the disturbing phenomenon of child sex abuse and the paedophile, 2) black boxing can be reflected in the trajectory of research and concomitant operationalisation of research questions and subjects, 3) there is a decrease of the discursive space in the media and the political arena, and 4) hasty or unscrutinised application in social policy can be expected. Finally, the envisioned institutional context of application of child care does not seem equipped to counterbalance the impetus for hasty application.

### Black boxing of the preference and the act

Despite the common use of the word ‘paedophile’ by all sorts of institutions, agencies, publics and (clinical or legal) experts, there are varieties in its interpretation.

From a clinical perspective, different types of child sexual abuse can be defined, with varieties in both the victim and the perpetrator. The child sex offence can be ‘situational’ – such as incest in the home – or ‘aggressive’ when the offence is committed by the ‘antisocial type’. In both cases, the offender does not necessarily have a primary sexual interest in children. In fact, half of child sex offences are perpetrated by individuals who do not meet diagnostic criteria for paedophilia (Blanchard et al. 2001, Maletzky and Steinhauser 2002). Other mental illnesses, psychological conditions or learning difficulties can have an influence on whether or not someone will engage in a sexual relation with a child (Bickley and Beech 2001, Howitt 1995). Thus child sexual abuse does not necessarily originate from sexual interest in children. The third main type is the child sex offence committed by a person who is primarily sexually attracted to children: the paedophile. Importantly, not all paedophiles commit these offences. Having a sexual interest in children *per se* does not sufficiently explain sexual offending against children (Seto 2010, Seto et al. 2006). Furthermore, paedophile refers to attraction to prepubescent children, meaning under the age of 11 (Blanchard et al. 2009).

Therefore, although paedophilia and child sexual abuse can overlap, they are not synonymous in the (forensic) clinic. Outside the realm of the clinical environment, however, these particular nuances are rarely considered (Rind et al. 1998). Legal interpretations necessitate a physical act, rather than the mere existence of a particular sexual preference (Harrison et al. 2010). Moreover, defining a child sex offence requires a legal interpretation on when the victim is a child or not. This relates to the societal perception of a young person’s capacity of giving *consent* to a sexual act, which is different from showing biological signs associated with puberty (Silverman et al. 2002, Thomas 2005). The age of consent has shifted significantly throughout history and differs considerably across cultures.

The public’s perception of paedophilia appears more in line with legal than clinical understanding (McCartan 2011). Much child sexual abuse is labelled as paedophilia, when from a clinical perspective this is not correct (Fagan et al. 2002, Seto 2010). When students were asked about typical traits of paedophiles in a study by McCartan (2010), most mentioned ‘sexually abusing children’ (68.6%), whereas only a minority indicated that this might not necessarily be the case (11.8%).

The linguistic issue is also highly political. Definitions of paedophilia across the different domains reflect moral choices which are rooted in what is deemed acceptable (Marecek and Hare-Mustin 2009). If child sexual abuse is equated to paedophilia in common understanding, then a prevention option targeting the paedophile, such as the paedoscan, is likely to receive wide support. Other types of child sexual abuse can then be obscured.

### Black boxing of the research trajectory

Brain imaging research into sexually deviant behaviour with child victims has been taking place from as early as 1967 (Kolarsky et al. 1967). Still, research into this topic has been – and remains – scarce. Interest in this field seems to be increasing somewhat, as the number of studies have been rising from 2000 onwards. This impetus seems related to the development of new neuroimaging modalities. Before 2000 mainly CT and EEG were used, after 2000 this changed to (f)MRI and also some PET studies. This coincided with a narrowing focus on paedophiles. Before 2000 there was interest in various types of perpetrators of child sexual abuse (e.g. Hendricks et al. 1988, Langevin et al. 1988), whereas after 2000 a limited focus on paedophilia can be observed (e.g. Habermeyer et al. 2013). The scarcity of this type of research in the face of high public concern is mentioned by various authors in this field, although reasons for it are seldom mentioned. Hughes (2007) ascribes it to discomfort with the topic. James Cantor recently claimed that there is also little funding for this field of research, and that far more money is being spent on detaining child sex offenders (van Eijck 2015).

Before 2000, the identified sources mostly concerned the unknown association between (structural) brain damage and sex offending (e.g. Graber et al. 1982). After 2000 the unknown neurobiological correlates of paedophilic interest took centre stage (e.g. Stoléru et al. 2013). The dominant curiosity of the scientific community therefore does not seem to be the identification of people with paedophilia, but to elucidate the brain mechanism underlying the disorder. However, although the scientists in general do not envision this option of identification, neurobiological correlates of paedophilia can also be used by others to attempt to identify paedophiles among job applicants.

Research efforts into paedophilia were mainly justified by the societal problem of child sexual offending and the concomitant public concern, especially from 2000 onwards. In these instances, paedophilia was equated with the act of child sex abuse (e.g. Moulier et al. 2012). Nevertheless, the relevance of their findings was predominantly perceived to be scientific (75%) and clinical (40%). Societal relevancy was acknowledged in a minority of the documents and related to criminal responsibility assessment.

The focus of neuroimaging research thus shows significant overlap with the societal framing of child sexual abuse. Research targets the paedophile brain and it is related to the ‘inevitable’ societal problem of child sex abuse; society views child sex abuse as a result of paedophilia. There is alignment between the curiosity of neuroscientists and what is societally perceived as a ‘relevant’ target (paedophiles), which is further legitimised by the awful reality of child sexual abuse. Importantly, this does not automatically make the science relevant to the nature of the societal problem of child sexual abuse. A scanning technology to determine paedophilic interest would fall short in identifying all potential child molesters, considering the difference in aetiology between for example paedophiles and incest offenders (Ames and Houston 1990). It would also be unable to discriminate between paedophiles at risk of (re)offending and non-dangerous paedophiles. However, paedophiles in general will be perceived as a relevant target if the association between paedophilia and child sexual abuse is and remains largely unquestioned.

Although scientists are studying neurobiological correlates for elucidating the aetiology of paedophilia, there is potential for premature application outside of the laboratory as the societal problem lies in the prevention of child sexual abuse, and not so much in the unsolved aetiology. This likelihood is also influenced by the perception of neuroimaging technologies.

There is a positive attitude of the general public to neuroscience crossing borders from the domain of basic research and the clinic, to other domains relevant to the phenomenon of child sexual abuse, such as security and justice. Brain-based options to prevent sexual delinquency cannot be separated from the perception of neuroscience as authoritative and prestigious (Pickersgill 2011a, b, Rose 2007) and the “conviction that science can deliver failsafe, and therefore just, legal outcomes where the law, acting on its own, might fall short” (Jasanoff 2006, p328; cited in Pickersgill, 2011a). This relates to the view of the biological mind as freely accessible and fundamentally truthful, and of neuroimaging as having revelatory powers (Littlefield 2009). In other words, neuroscience is seen as capable of objectively measuring deviation, from which evaluations and management of behaviours can be deduced (Littlefield 2009). Pickersgill (2011a) has argued that the idea that neuroscience travels to other domains is not only supported, but that it is even imagined as epistemologically superior to the domain of law.

Re-use of preliminary findings of neuroscientists in other domains is thus probable. Moreover, as research questions are conceptualized in terms of paedophilic interest, neuroimaging research is likely to inform child protection policies regarding paedophiles and thus leading it away from other types of child sex offenders. Scrutiny is thus necessary when there are calls for the development of a paedoscan to prevent sexual abuse of children.

### Decrease of the discursive space in the media and political arena

In the media, paedophiles are often stereotypically depicted as violent criminals (Kitzinger 2002). Analysis of media representations of sex crimes has also shown that coverage typically ignores wider issues in favour of sensationalism, characterized by a lack of discernment between different types of sexual offences (Greer 2013, Silverman and Wilson 2002, Soothill and Walby 1991). Stories on paedophiles can be found in print, on television and in films, illustrating that it is a high-profile public interest issue in Western society (McCarten 2010).

It has been argued that media attention on the paedophile resembles that of a ‘moral panic’, out of proportion to the reality of the risk (Critcher 2002, Silverman and Wilson 2002, Thomas 2005). Moral panics revolve around a perceived threat to something held dear and almost sacred by society, for which an event, group or subculture is scapegoated (Cohen 2002). Public fears and fantasies are projected onto this ‘folk devil’. The media is seen as a key player in the development and maintenance of moral panics as disseminators of moral indignation. Stan Cohen (2002) has argued that moral panics emerge at times of wider social unease and rapid change, resulting in anxiety. However, child sex abuse is not a recent or rapidly changing phenomenon. It may be that the paedophile is scapegoated because of wider anxieties around sexualisation of the child, as the increased attention to paedophilia has coincided with a growing and accepted sexualisation of children taking place in the media, fashion and the cosmetics industries (Silverman and Wilson 2002).^4^ For example, the borders between women and girls have been blurring rapidly: women are pubic hair free, and girls are dressing like women (Silverman and Wilson 2002). To regain control, folk devils are singled out as a target in an all-pervasive demand that ‘something should be done’ (Cohen 2002). This suggests that the highly mediatized issue of the paedophile is more than just a media frenzy: it sets the stage for certain kinds of action.

For highly mediatized and politicised issues, a close association has been observed between media reporting and activities in the political arena (McCallum 2013). Kitzinger (2002) demonstrated that at the height of media attention to paedophilia in the UK, a perilous entwinement between media and policy-makers existed and it was unclear whose activities drove whose agenda. Literature on the mediatization of politics argues that media content can be governed by political logic and vice versa (Strömbäck 2008). Furthermore, in the speeding up of the media, as illustrated by the spread of digital ‘real-time journalism’, politics also seems to have accelerated (Rosa 2005). Political decision-makers are somehow ‘forced’ to react quickly to stories in the media.

This is a far cry from the potential positive contribution the media could have. Rather than providing a forum for public dialogue, it appears to increase the risk for hasty or bad decisions (Helms 2012, Kitzinger 2002). Moreover, media coverage on (child) sex offenders has been observed as a source of political capital. Moreno (1997) describes the use of stereotypical depictions of the sex offender in the media for political and media gain. Getting tough on child sex offenders has been shown as a way to help ensure re-election (Meloy et al. 2013). It can be advantageous to a political figure to be seen as doing something against paedophiles, such as advocating the application of the paedoscan in child care institutions.

Addressing the topic of paedophilia can be very treacherous in the political arena. On taboo topics, positions are quick to polarize: you are either against paedophiles, or you are with these ‘evil monsters’. Opening the debate to wider issues is a quick way to jeopardize one’s position as a legitimate politician (or scholar for that matter). Some evidence points towards paedophiles as elicitors of disgust (Lynch 2002). It can therefore be dangerous to plead for reticence in the introduction of a tool such as the paedoscan, as you can become ‘contaminated’. Your political intentions can become suspect and you can be conceived as one of ‘them’.

Deviation from mainstream rhetoric is thus dangerous, because of mechanisms that suppress other types of discourse. As disgust is an unreasoned aversion that requires no further explanation, in the sense that it is cognitively perplexing, wider utilitarian reasoning about the pros and cons of a technology - such as the paedoscan - appears impossible (Tetlock 2000). It is important to recognize that a cultural phenomenon such as disgust is not restricted to ‘the general public’, but affects all societal groups: also scientists, technology developers, policy-makers and politicians. This is a great obstacle to responsible technology development and embedding strategies that depart from the idea of wider deliberation. Political figures are likely to be reluctant to instigate or engage in such wider deliberation, especially if this would be picked up by the media. With the application of the paedoscan, civil liberties of paedophiles are at stake as they would be restricted in their freedom because of their thoughts. Furthermore, a disproportionality argument can be made as the paedoscan is not likely to screen for other types of child sex offenders. However, in a context of moral outrage and demands for ‘something to be done’, it would be difficult to start such a deliberative conversation in the event of public demands for a paedoscan.

### Hasty application in social policy

When convicted child sex offenders are released in some Western countries, legislation often enforces involuntary treatment in addition to other provisions for ‘special’ arrangements for sex offender management, such as preventive detention, community notification and residency restriction laws (Wright 2008). These special measures go beyond traditional parole and probation structures, and are typically *social* policies rather than *penal* ones. One rationale for this is that these laws and regulations are aiming to protect the public and decrease societal distress. But civil regulations also circumvent stricter constitutional requirements in criminal law, in the restriction of freedom and privacy of sex offenders (Hamilton 2010). This restriction of civil liberties is beyond what any other type of criminal offender experiences, including those who have committed murders or have been engaged in armed robbery (Pickett et al. 2013).

In the Netherlands, there are no such special legal arrangements for sex offenders. Nevertheless, they are treated as a special type of offender. There is a higher focus on prevention for this type of offender as they are more likely to receive an additional conditional sentence than non-sexual crime offenders on top of their unconditional imprisonment (Leuw et al. 2004). After conviction, they can be subjected to polygraph testing. In the Netherlands the polygraph is not used in police investigations and has been rejected as evidence in court (Meijer and van Koppen 2008). However, it *is* used for the management of child sex offenders and not for any other type of non-sexual crime offender (Buschman et al. 2010). In the US and the UK, the polygraph is also put to this use (Meijer and van Koppen 2008, Petrunik 2002).

The polygraph can be considered as a kind of precursor technology to the paedoscan. Brain-based detection technologies, such as fMRI, are compared to the polygraph according to Littlefield (2009). She contends that new brain-based detection technologies are likely to become marketable especially *because* of what they share with polygraphy (Littlefield 2009). It is thus relevant to consider how the polygraph is used in current sexual offender management, to see what it can teach us about the potential use of the paedoscan in social policy.

In January 2014, British Members of Parliament were “expected to clear the way for the introduction of compulsory lie detector tests”, to monitor “serious sex offenders who have been released into the community after serving their prison sentence”, following a “successful pilot scheme” despite “[c]ontinuing concerns about the reliability of the tests and misinterpretation of the results” (Travis 2013, Watt 2013). The pilot was deemed successful as offenders were found twice as likely to disclose contacting a victim or breaching terms of their release during interviewing when connected to a polygraph. Increased disclosure of (child) sex offenders faced with the polygraph has also been found in other studies in Europe and the US (Ahlmeyer et al. 2000, Buschman et al. 2010, Emerick and Dutton 1993, English et al. 2003, Grubin et al. 2004, Wilcox and Sosnowski 2005). However, this is probably due to the sex offenders’ belief that the polygraph is capable of detecting deception, and not to the accuracy of the polygraph itself. This effect is known as the ‘bogus pipeline’ effect (Meijer et al. 2008, Roese and Jamieson 1993). Disclosures may not have been accurate. The only option is to compare information obtained through polygraph testing with what is written in the offender’s files (Meijer et al. 2008). Newly disclosed information is considered a success, but as the ground truth is unknown, the accuracy of the disclosed information is also unknown. Diagnostic accuracy of fMRI is (still) low, as there is low sensitivity and specificity for screening on an individual level (see, for example, Feigenson 2006). However, it could be that this will be overlooked when applied to paedophiles, as is happening now for the polygraph.

If a sub-par technology such as the polygraph is condoned for child sex offenders in social policy, it is conceivable that a similar future awaits contiguous brain-based technologies for the same offender group. Considering the increased focus on prevention policy for the perpetrators of child sexual abuse compared to other offenders, there appears to be a fertile ground for the paedoscan to emerge. The internationally condoned use of technologies impressive at face-value – technologies that are not so much accurate but rather have an ability to persuade – in social policies targeting child sex offenders indicates that hasty application of a brain-based detection technology for paedophiles is quite probable. Similar to its precursor technology, the polygraph, it is plausible that a lack of diagnostic accuracy is accepted for the paedoscan in the eagerness to make a difference in the harsh reality of child sexual abuse.

### Problematic embedding in formal care settings

As in other Western countries, the number of children attending a child care centre has increased rapidly in the Netherlands (Vermeer et al. 2008). At the same time, the quality of child care centres in the Netherlands has significantly decreased since the 1990s (Vermeer et al. 2008). The quality of child care is perceived to suffer from the low status of this profession in Dutch society with concomitant low wages (Mahon 2002).

Media reporting on cases of child sexual abuse in child care during the past decades may give rise to the perception that children in child care settings are at increased risk of child sexual abuse (Colton et al. 2012). However, child sexual abuse occurs much more frequently inside the home than in child care services (Schumacher and Carlson 1999). Nevertheless, any setting where children are present can attract perpetrators who use their work as a cover for sexually abusing children (Sullivan and Beech 2002).

Prevention efforts of child sexual abuse in child care settings can entail proofing of the child care practice itself (Sullivan and Beech 2002), for example, by enforcing a policy that child care professionals are never alone within a group of children and that doors cannot be locked. A second strategy is to identify problematic individuals, for example by screening applicants.

One way to predict potential for child sexual abuse perpetration is to look at past criminal history. However, few perpetrators have prior arrests for sexual behaviour (Schumacher and Carlson 1999). Reliance on criminal background checks is thus insufficient to prevent child sexual abuse by child care workers. For jobs involving dealings with vulnerable people, Dutch employers are required by law to ask applicants for a Certificate of Good Conduct. This is a document issued by the Dutch Ministry of Security and Justice which declares that the applicant did not commit any criminal offences relevant to the nature of the job.^5^

If brain-based detection could be used to identify potential paedophiles among job applicants of child care facilities, this would at best be a statistical prediction. Could this still be of added value? In one of the sparse studies on applicant screening among UK child service organisations it transpired that hiring decisions are largely based on skill assessments and ‘gut feelings’ regarding the applicant’s motivation to work in this particular setting, in combination with over-reliance on background checks (Price et al. 2013). Acquiring a job in these organisations will not prove difficult in the absence of a criminal record and when the applicant presents well. However, can applicants be forced to undergo such scans?

In the UK, the screening of job applicants by means of neuroimaging is legal, when employers are able to prove that it is related to performance and safety issues of the job (Shivers 2004). In the US, it depends on consensus on the reading of the Federal Employee Polygraph Protection Act of 1998 (FEPPA) (Simpson 2008). There is a debate on whether employees and applicants can be assessed to detect lying with brain-based detection technologies. Generally, the use of *polygraph* examinations is barred by the FEPPA. What is understood in this act as a ‘polygraph’ could also include brain-based lie detectors. However, this debate will continue as long as there is no binding legal interpretation on this matter. In the Netherlands, there are legislative restrictions concerning the medical testing of job applicants (Wet op de medische keuringen, Wmk). For jobs in which it is essential that an employee fulfils certain medical requirements to prevent endangerment to himself or others, employers are allowed to inquire after, and test for, specific medical conditions during the application procedure. The tests are performed by independent professionals who are legally bound to confidentiality and can only disclose whether the applicant has failed or passed. Moreover, as much as possible, there should be an evidence base for the test. For a brain-based detection technology to be adopted as a predictive screening test, it should have a similar or higher diagnostic value than other existing tests.^6^ It thus requires a certain degree of consensus on the relative evidence base.

There is a dearth of existing reliable and valid instruments for the child care institutions. Abel et al. (2012) have developed a questionnaire for this setting, called Diana Screen®, but so far no prospective studies have been performed (Wurtele 2012). A paedoscan might thus not be ‘ready’ in the eyes of the scientist, but could still outperform other tests in the absence of strong competitors. Furthermore, the determination of what is evidence-based and what is not, is not only informed by a ‘material reality’. Rather, ‘closure’ on the evidence-base is informed by social processes of human work and cultural notions on both the science and the envisioned application domain. It is conceivable that closure on the evidence-base of a brain-based detection technology can be achieved sooner for this particular application than for others, because of the felt urgency to ‘do something’.

(Dutch) child care institutions do not seem equipped to offer resistance to hasty application of the paedoscan. There are no strong competitors for the paedoscan as a tool, and there is little experience with screening for deviant sexual motivations to apply for a position in a child care facility. The future use of a paedophile scan as a medical test in the application process in child care settings is thus conceivable. However, to prevent child sexual abuse, it is necessary not only to identify paedophiles who are likely to (re)offend, but also (prospective) child sex offenders without a sexual preference for children. Focusing on paedophilic interest alone will likely decrease efficacy. Branding a child care facility as paedophile free would give a false sense of security to parents conflating paedophilia with child sexual abuse.

## Discussion

The policy problem of the prevention of child sexual abuse in child day care centres by scanning the brains of job applicants appears a moderately structured or even a structured problem, because of the black boxing of the problem definition, potential solutions and development trajectories. For example, black boxing narrows down which directions are considered as viable sources of solutions. Paedophiles are singled out as potential child sex offenders and their paedophilic interest is seen as the main driver behind offending behaviour, rather than other behavioural characteristics (such as a decreased impulse control) or environmental factors (social isolation).

In the debate surrounding the development of the paedoscan, it is highly likely that the value of privacy will be masked while ‘producing’ potential child sex offenders. As a paedoscan that focuses on romantic or sexual feelings towards children – rather than sexual acts on children and associated behavioural characteristics that make such an act more likely – a specific suspect gets created, one that corresponds with the cultural idea of the typical child sex offender, namely the paedophile. Paedophiles cán be sex offenders but the reverse does not prove true. The associated feeling of disgust towards paedophiles seems to make it easier to disregard the value of privacy for this produced suspect. In a similar fashion, the value of ‘the good life’ and autonomy of a person with paedophilia will tend to get neglected in the debate because of cultural notions on paedophiles and feelings of disgust. Paradoxically, the value of child safety and the autonomy of parents in making informed decisions about their child’s safety are also likely to get (at least partly) masked in the innovation process of the paedoscan. The targeting of the paedophile shields from view other potential sex offenders. Moreover, it has been found that paedophiles who are excluded from society are more likely to offend. As such, feelings of disgust seem able to mask the pivotal value of child safety. Furthermore, it is likely that in the development and the deployment of the paedoscan such as discussed in this paper, parents will not get the information they need to make informed choices: 1) They may think that the only danger comes from (strange) paedophiles and forget about dangers from within their circle of family and friends, and; 2) they may be convinced to think that child care institutions which deploy the scans are safer than those who don’t, and may therefore be less aware of warning signs.

Because of the masking of facts and values there seems to be little necessity for a problem structuring approach via deliberation and produces early closure. However, when early closure is the result of a taboo topic, which constrains critical reflection, there is reason for caution. Unstructured problems can then be perceived as doable (moderately) structured problems, for which straightforward solutions are thought to exist, as we have shown here to be case for the paedoscan if it were to be developed. Early closure lays the foundation for a problematic embedding of such a prospective paedoscan, as interpretations outside of the mainstream rhetoric are foreclosed.

It is not without repercussions to try to widen the conversation on problem statements and technology purposes, no matter how sound the argument, when early closure is induced by taboo. The most dangerous effect is that of contamination by the elicitor of disgust, the paedophile. At the same time, taboo rhetoric can be used to gain public support. It thus seems easier to go along with demands for (or to propose) new technologies and tougher policies. Hasty application is then probable, and unlikely to produce the responsible embedding of such a technology. Trying to slow down development, or trying to prevent potential abuse of the technology, increases the risk of the contamination effect.

### Developing the RRI framework: four lessons

What does this mean for the emerging RRI community? First of all, it implies a need for alertness to signs of early closure, such as hyper alignment on problem statements and purposes between otherwise very different stakeholders. In the case of the paedoscan, early closure should be anticipated because of the presence of taboo topics. For non-controversial emerging technologies, early closure can also happen in the presence of powerful stakeholders. For example, in a study by Elberse et al. (2011), a high degree of alignment was observed between users of congenital heart disease care and cardiologists during a dialogue which aimed to include patient perspectives into clinical research. In this particular instance, the cardiologists formed a rhetorical coalition and subordinated the rich experiences of the care users by reframing these topics in such a way that they became part of an umbrella term that matched the jargon of the experts. Strikingly, the care users experienced the dialogue as satisfactory, in the sense that they felt taken seriously and contributed useful knowledge. This indicates that RRI process facilitators cannot rely on stakeholder dissatisfaction as a subtle signpost of early closure during singular engagement activities. Whether early closure is induced by taboo or by powerful stakeholders exerting their dominance, the RRI community should be aware of this phenomenon as it can preclude a genuine dialogue. Moreover, these observations also provide a warning for the emerging RRI community that a process of engagement with all relevant and different stakeholders, as argued for in RRI literature (see for example Owen et al. 2012), does not necessarily lead to an outcome in tune with the relevant and different societal values as masking of value diversity can take place in dialogues and society alike.

Processes of responsible research and innovation, therefore, do not only require a skilled facilitator of engagement activities; it also needs a critical reflector who is alert to signs of early closure – for which taboo is one of the potential sources – and who initiates critical reflection on early closure. This may involve undertaking a multidisciplinary study of ethical, legal and social aspects (ELSA) of the phenomenon for which early closure is observed, parallel to the engagement process. In other words, the second lesson to RRI is to adopt multiple methods to avoid early closure the field of RRI instead of relying on the one method heralded most; engagement. Although this suggests an important role for ELSA studies within the framework of RRI, this also touches upon the normative position of the critical reflector adopting an ELSA approach. If there is such high alignment, when can we still speak of a value conflict in need of resolution, and from which position can the critical reflector decide that it is so? Arguing from the framework of RRI such an intervention would be necessary for the sake of ‘reflexivity’, as high reflexivity points towards a sound RRI process.

Third, there is a challenge to develop participatory tools appropriate for topics that elicit the emotion of disgust. Participatory approaches such as the (re)framing of issues (Schon and Rein 1994) have shown their merits for public deliberation on emerging technologies (Arentshorst et al. 2016, Edelenbosch 2014, Roelofsen 2011). However, the dominant discourse around the issue of paedophilia and mechanisms of contamination are at odds with conditions for ideal speech (Habermas 1981) characteristic to such approaches, namely the willingness of participants to change their minds when presented with a better argument (openness), and putting social hierarchies (equality) and power relationships (absence of power play) in the background. Moreover, Habermasian discourse ethics in participatory approaches has been criticized for neglecting the role of emotions, such as anger, fear or disgust, in favour of ‘neutral’ rational discourses. This is seen as problematic because as emotions are central to how stakeholders deal with information (Buijs and Lawrence 2013, Fischer 2009). Or, as Martha Nussbaum (2003) puts it, there is intelligence in emotions. The way forward appears to us to make participatory tools more sensitive to emotions, however not to the emotion of disgust. Instead, it appears to us that it is wiser to redirect or circumvent the emotion of disgust to other emotions. Our thought process is the following. Emotions are important to consider because of their connection to basic thought processes and because they contain a form of tacit intelligence (Nussbaum 2003). Disgust is indeed intrinsically linked to thought processes as it stops them (Tetlock 2000), but the emotion of disgust is unlikely to constitute a form of tacit intelligence on which can be relied as a guide for public deliberation or practice. Quoting Martha Nussbaum’s seminal work on disgust in the public domain of the law, the thought-content of disgust is “typically unreasonable, embodying magical ideas of contamination, and impossible aspirations to purity, immortality, and nonanimality” and “disgust has been used throughout history to exclude and marginalize groups or people who come to embody the dominant group’s fear and loathing of its own animality and mortality” (Nussbaum 2004, 14). This should make us sceptical on expecting a constructive contribution of this emotion within public deliberation processes. However, it may be possible to redirect disgust by tapping into other pertinent emotions associated with the issue of child sexual abuse, such as anger, fear, hope, grief and love. For example, one can feel anger at an assault or damage, fear for the safety of well-being of a child, grief over the loss of dignity, have love for other beings and hope for resilience. Therefore, more experience is needed in adapting participatory methods in such a way that these emotions are not treated as inherently irrational. This could be done, for example, by explicitly looking for exploring emotional frames (see Buijs and Lawrence 2013) or by making emotions a topic of conversation in (non-emotional) frames. For the latter, a facilitator may ask questions what emotions the participants are feeling during the exploration of a certain frame. This conversation can be deepened by inquiring into what degree participants are feeling an emotion, the beliefs associated to said emotion are and the ‘reasonableness’ (Nussbaum 2004) of certain degrees of emotions and beliefs.

Fourth, facilitators of RRI processes need to reflect on the normativity of engaging people in wider deliberation of taboo subjects considering the societal repercussions on the participants (and possibly on the facilitator him/herself). We also suggest that a task (or even a duty) lies here for the emerging RRI community to contribute to agenda setting for research endeavours into taboo subjects and to opportunities for funding of unpopular research, especially if scientists are willing but as yet unable to do this type of work. This is related to the recognition that ‘innovation’ does not build upon the reiteration of the same ideas, but requires critical discourse, so that alternatives can be explored.

### Responsible development and embedding of the paedoscan?

The responsible development and embedding of the paedoscan starts by unpacking the phenomena of child sexual abuse, the person with paedophilic interests as well as how these are interrelated. This requires a safe place created by a skilled facilitator where elaboration on the relevant aspects can occur. Enough participation of stakeholders with differing views is necessary to allow for expansion of the discursive space, but the space should be kept small enough to mitigate spill over contamination effects. Facilitators should be aware of fact and value masking due to the presence of triggers of disgust, and try to redirect disgust to other salient emotions, such as anger, fear, sadness and love. People with paedophilia are one of the groups to be included in the deliberation process: maybe not in multi-stakeholder dialogue events, but rather in homogeneous meetings of which the results can be fed into subsequent deliberations (and vice versa).

During such deliberations, it may turn out that the problem of child sexual abuse in the context of out-of-home child care is in need of another (perhaps low-technology) solution than the paedoscan. In other words, it is possible that the outcome of an inclusive deliberation process is that responsible development and embedding of a paedoscan is impossible, and another option is favourable. For example, one that targets all potential child sex offenders, and not merely those people with a paedophilic interest. This type of alternative outcome is more likely to be achieved if scientists and developers of competing sources of solutions are included as participants (de Jong et al. 2016). Or it could be that viable trajectories for a paedoscan could become apparent through the deliberations. In either case, this will yield a shared point on the horizon to work towards. This is important as demands for action, corresponding with the narrow conception of the problem, can arise at any given moment. It can serve as a strong competitor to other ideas that are less likely to be responsible.

This thought experiment shows the importance of acknowledging the diversity and power relations among stakeholders to avoid the pitfall of masking potential fact and value conflicts. It provides a compelling case for the concepts of value/fact diversity masking and early discursive closure as a new avenue for RRI research and for RRI researchers and practitioners to pay attention to early closure in RRI processes. Moreover, it yields some suggestions on how to go about it in the case of taboo topics. Nevertheless, further research on fact/value diversity masking and early discursive closure can affect RRI processes is needed, as well as empirical studies in which methods to cope with early discursive closure are explored, so that lessons can be drawn for the emerging framework of RRI.

## Appendix: Search query

The search query was constructed by the combination of the following three building blocks:

1. Technique of neuroimaging (NI): “brain scan*” OR neuroimaging OR “brain imaging” OR “brain image” OR positron OR tomography OR “Magnetic Resonance Imaging” OR “functional Magnetic Resonance Imaging” OR electroencephalography OR “deep brain stimulation” OR neurostimulation OR “brain machine interfac*” OR “transcranial magnetic stimulation” OR spect OR pet OR TMS OR mri OR fmri OR eeg OR spectroscopy OR meg OR scintigraph*
2. Perpetrator: Sex offender (from a criminal perspective); Pedophile/paedophile (from a clinical perspective)
3. Act: Child abuse

The search strings were adapted to the respective databases.

A. PubMed
  NI: {Text Word]“brain scan*” OR “brain scan” OR “brain scans” OR neuroimaging OR “brain imaging” OR “brain image” OR positron OR tomography OR “Magnetic Resonance Imaging” OR “functional Magnetic Resonance Imaging” OR electroencephalography OR “deep brain stimulation” OR neurostimulation OR “brain machine interface” OR “transcranial magnetic stimulation” OR spect OR pet OR TMS OR mri OR fmri OR eeg OR spectroscopy OR meg OR scintigrap*
  AND at least one of the following:
  [Text Word] “sex offender”
  [Text Word] dunkelfeld
  [Text Word] (paedoph* OR pedoph* OR paraphi* OR pedosex* OR hebephi* OR pedohebephi*)
  [Text Word] (“sex offence” OR “sex offences” OR “sex offender” OR “sex offenders” OR “sex offense” OR “sex offenses”) AND (child OR children OR boy OR girl OR prepubescent OR boys OR girls)
  [Text Word] (“sexual offence” OR “sexual offender” OR “sexual offense” OR “sexual offences” OR “sexual offenders” OR “sexual offenses”) AND (child OR children OR boy OR boys OR girl OR girls OR prepubescent)
  [Text Word] ((“child abuse” OR “child abuser”) AND sex)
  [Text Word] (“Child molester” OR “statutory rape” OR “sexual predator”)
B. Web of Science
  NI: [Title] or [Topic]: (“brain scan*” OR “brain scan” OR “brain scans” OR neuroimaging OR “brain imaging” OR “brain image” OR positron OR tomography OR “Magnetic Resonance Imaging” OR “functional Magnetic Resonance Imaging” OR electroencephalography OR “deep brain stimulation” OR neurostimulation OR “brain machine interface” OR “transcranial magnetic stimulation” OR spect OR pet OR TMS OR mri OR fmri OR eeg OR spectroscopy OR meg OR scintigrap*)
  AND at least one of the following in [Title] or [Topic]:
  “sex offender”
  “dunkelfeld”
  (paedoph* OR pedoph* OR paraphi* OR pedosex* OR hebephi* OR pedohebephi*)
  (“sex* offenc*” OR “sex* offend*” OR “sex* offens*”) AND (child OR children OR boy OR girl OR prepubescent OR boys OR girls)
  (“child abuse*” AND sex)
  (“Child molest*” OR “statutory rape” OR “sex* predator*”)
C. ScienceDirect
  NI: TITLE-ABSTR-KEY ((“brain scan*” OR “brain scan” OR “brain scans” OR neuroimaging OR “brain imaging” OR “brain image” OR positron OR tomography OR “Magnetic Resonance Imaging” OR “functional Magnetic Resonance Imaging” OR electroencephalography OR “deep brain stimulation” OR neurostimulation OR “brain machine interface” OR “transcranial magnetic stimulation” OR spect OR pet OR TMS OR mri OR fmri OR eeg OR spectroscopy OR meg OR scintigrap*))
  AND at least one of the following in TITLE-ABS-KEY
  “sex offender”
  “dunkelfeld”
  (paedoph* OR pedoph* OR paraphi* OR pedosex* OR hebephi* OR pedohebephi*)
  (“sex* offenc*” OR “sex* offend*” OR “sex* offens*”) AND (child OR children OR boy OR girl OR prepubescent OR boys OR girls)
  (“child abuse*” AND sex)
  (“Child molest*” OR “statutory rape” OR “sex* predator*”)
D. PsycINFO
  NI: All Text ((“brain scan*” OR “brain scan” OR “brain scans” OR neuroimaging OR “brain imaging” OR “brain image” OR positron OR tomography OR “Magnetic Resonance Imaging” OR “functional Magnetic Resonance Imaging” OR electroencephalography OR “deep brain stimulation” OR neurostimulation OR “brain machine interface” OR “transcranial magnetic stimulation” OR spect OR pet OR TMS OR mri OR fmri OR eeg OR spectroscopy OR meg OR scintigrap*))
  AND at least one of the following in All Text
  “sex offender”
  “dunkelfeld”
  (paedoph* OR pedoph* OR paraphi* OR pedosex* OR hebephi* OR pedohebephi*)
  (“sex* offenc*” OR “sex* offend*” OR “sex* offens*”) AND (child OR children OR boy OR girl OR prepubescent OR boys OR girls)
  (“child abuse*” AND sex)
  (“Child molest*” OR “statutory rape” OR “sex* predator*”)

## Abbreviations

CT: Computed tomography
EEG: Electroencephalography
ELSA: Ethical, legal and societal aspects
FEPPA: Federal Employee Polygraph Protection Act of 1998
fMRI: Functional magnetic resonance imaging
MRI: Magnetic resonance imaging
PET: Positron emission tomography
RRI: Responsible Research and Innovation
Wmk: Wet op de medische keuringen

## Footnotes

1 Neurosciences in Dialogue focuses on the responsible development and use of neuroimaging in three application domains - health care, education and justice & security - and involves researchers and societal stakeholders in an interactive learning process to stimulate developments towards shared desirable applications with few, or at least manageable, negative impacts.

2 In December 2010, a Latvian born child day care worker was arrested in Amsterdam for producing and distributing child pornography. He turned out to be one of the most prolific European sex offenders of all time (Jacobs and Blitsa 2012; Sterling 2012). National shock and public outrage ensued when case specifics emerged. The ‘Monster of Riga’, as he was called, possessed terabytes of child pornographic images and movies, some of which he produced himself, and admitted to the abuse of 83 young children entrusted to his care, either as a day care employee or freelance babysitter. The victims ranged in age from 19 days to a few years old.

3 A “black box” refers to an object in which inputs are transformed into predictable outputs, but the way it does this cannot be seen from the outside (MacKenzie 2005). The inside of the black box is treated as mere technical details, therefore to be ignored. One example of a black box one is that of recognized expertise. On a daily basis, we place our lives in the hands of surgeons or pilots, with whom we have no personal experience. Technologies and artefacts are often treated as black boxes, although various readings in the field of science-and-technology studies have shown how technologies cannot be treated as merely technical but also as products of constitutive social relations (Law and Hassard 1999).

4 This is related to the ideology of the child as innocent, in which recognition of the child’s sexuality is withheld (see for example Kitzinger 1988 and Kleinhans 2002).

5 In the case of the Latvian child sex offender in the Dutch scandal there were previous convictions, but these were unknown as they took place outside of the Netherlands.

6
www.aanstellingskeuringen.nl/publicaties/adviezen/2009/2009_07.aspx
